# CpG Island Methylation in Colorectal Cancer: Past, Present and Future

**DOI:** 10.4061/2011/902674

**Published:** 2011-04-12

**Authors:** Karen Curtin, Martha L. Slattery, Wade S. Samowitz

**Affiliations:** ^1^Department of Internal Medicine, University of Utah Health Sciences Center, Salt Lake City, UT 84112, USA; ^2^Department of Pathology, University of Utah Health Sciences Center, Salt Lake City, UT 84112, USA

## Abstract

The concept of a CpG island methylator phenotype, or CIMP, quickly became the focus of several colorectal cancer studies describing its clinical and pathological features after its introduction in 1999 by Toyota and colleagues. Further characterization of CIMP in tumors lead to widespread acceptance of the concept, as expressed by Shen and Issa in their 2005 editorial, “CIMP, at last.” Since that time, extensive research efforts have brought great insights into the epidemiology and prognosis of CIMP+ tumors and other epigenetic mechanisms underlying tumorigenesis. With the advances in technology and subsequent cataloging of the human methylome in cancer and normal tissue, new directions in research to understand CIMP and its role in complex biological systems yield hope for future epigenetically based diagnostics and treatments.

## 1. Introduction

In the October 29, 2010 issue, Science turned the spotlight on epigenetics—a term that encompasses histone modification, nucleosome location, noncoding RNA, and DNA methylation. Epigenetic processes do not involve changes to DNA sequence but rather are self-propagating molecular signatures that are potentially reversible, unlike changes in genetic information [1]. DNA methylation is the most widely studied epigenetic marker [2]. The discovery of global DNA hypomethylation in human tumors was followed by the identification of hypermethylated tumor-suppressor genes and recently, inactivation of microRNA (miRNA) by DNA methylation also has been described [3–5]. Growing interest in epigenetic systems stems from an inability to determine causative genetic variants in many disorders. It is hoped that a better understanding of these systems may provide insight into our understanding of complex diseases such as cancer. 

Approximately half of all protein-encoding genes in the human genome contain CG-rich regions in their promoters or CpG islands. Aberrant DNA methylation, in the form of hypermethylation of CpG islands, results in repression of transcription in tumor suppressor genes. For example, inactivation of the mismatch repair gene *MLH1 *by promoter methylation is the molecular basis for microsatellite instability in sporadic microsatellite unstable colon cancers [6]. This phenomenon of tumor alteration via epigenetic silencing associated with dense hypermethylation of CpG islands, and their complex interplay with modifications in histone structure, provides an alternate mechanism to genetic inactivation of tumor suppressor genes via loss or mutation [2]. The presence of widespread CpG island methylation in a tumor is termed the CpG island methylator phenotype, or CIMP, and this paper is focused on this specific aspect of epigenetics.

## 2. CIMP in Colorectal Cancer

The role of CIMP in colorectal carcinogenesis was originally postulated in 1999 by Toyota et al. [7]. Their pioneering study distinguished between age-related and cancer-related methylation and defined CIMP in terms of the latter. Recognition of CIMP as a phenomenon in colorectal cancer is relatively recent; more than a decade earlier, in 1988, Vogelstein and colleagues developed a model that hypothesized that colorectal neoplasia occurs from a series of genetic alterations that includes activation of oncogenes and inactivation of tumor suppressor genes [8]. The concept of an epigenetic etiology in cancer introduced in the late 1990s was met with some controversy and resistance in the field of carcinogenesis [6, 9, 10]. However, the existence of CIMP has since gained wide acceptance, as the epidemiology characterizing this epigenetic alteration and its utility in understanding carcinogenic pathways support its significance in colorectal cancer biology [11–14].

**Figure 1:**
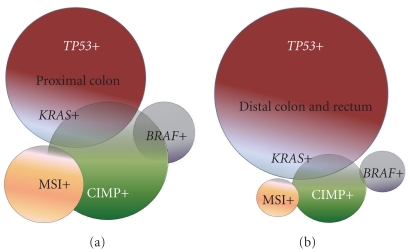
In colorectal cancer, CIMP+ occurs more frequently in tumors of the proximal colon (Figure 1(a)) and less frequently in tumors of the distal colon and rectum (Figure 1(b)). An approximate distribution of genetic and epigenetic tumor alterations is shown.

Most sporadic microsatellite unstable colon tumors are CIMP positive, whereas CIMP is uncommon in Lynch syndrome-associated cancers which exhibit microsatellite instability (MSI), indicative of distinct underlying molecular processes [13, 15]. Based on a number of relatively large case-control and prospective cohort studies, ~30–40% of sporadic proximal-site colon cancers are CIMP positive, compared to 3–12% of distal colon and rectal cancers [16–21] as illustrated in Figure 1. Thus CIMP is significantly more frequent in tumors of the proximal colon, and this is independent of MSI status. CIMP is also associated with *BRAF *mutations in both microsatellite stable and unstable colon cancers [11, 18, 20]. CIMP is observed in proximal hyperplastic (serrated) polyps, suggesting this lesion may be a precursor to unstable tumors (and perhaps stable tumors) in the CIMP high pathway [22]. Once thought to lack potential for malignant progression, hyperplastic polyps are now considered to represent a heterogeneous group, most of which harbor *BRAF* mutations and some of which exhibit epigenetic alterations (both uncommon in colorectal adenomas). A subset of hyperplastic polyps has been defined by architectural features and renamed sessile serrated polyps (or sessile serrated sessile adenomas). Most of these polyps are right-sided and many show CIMP, supporting the notion that they may be a precursor lesion for CIMP high tumors [23, 24]. 

Some case-control and cohort studies have reported a poor prognosis associated with CIMP in combination with microsatellite stable tumors [19, 25–27], although this may reflect the co-occurrence of *BRAF* V600E mutations, which have been associated with significantly poorer survival in colon cancer [28, 29]. Relatively minor effects of CIMP on prognosis suggest that the effect of mutations in *BRAF* on survival in stable tumors is not dependent on CIMP [16, 28]. Indeed, Ogino et al. reported that CIMP-high appears to be an independent predictor of a *low* colon cancer-specific mortality [30]. These results suggest the need for a large sample size to determine the relative contributions of *BRAF* and CIMP on prognosis.

## 3. Characterization of CIMP

In contrast to the relatively straightforward determination of MSI tumor status, a consensus as to the optimal panel of CpG sites for CIMP determination is only starting to take shape (Table 1). Different panels may yield slightly different results, although a strong relationship to the presence of a *BRAF* V600E mutation is consistently observed with all panels. The so-called “classic” panel of Park et al. utilized to assess CIMP status consists of CpG sites in *MLH1*, *CDKN2A* (p16), and methylated in tumors (MINTS) 1, 2, and 31 [31]. It has been suggested that there are two general types of CIMP in sporadic tumors: CIMP high, related to *BRAF* mutations and *MLH1* methylation; and CIMP low, related to *KRAS* mutations [12, 32, 33]. Tumors characterized as CIMP positive (CIMP+) based on the classic panel include both CIMP high and CIMP low categories; therefore, a subset of CIMP+ associates with *BRAF* and another with *KRAS* mutations, somewhat surprising given mutations in these genes are typically mutually exclusive since both are members of the ras signal transduction pathway [6, 11]. 

 Based on a systematic screen of 195 CpG sites and an unsupervised two-dimensional cluster analysis, Weisenberger et al. proposed a robust alternative to the classic panel to classify CIMP+ tumors consisting of *CACNA1G, IGF2, NEUROG1, RUNX3, *and *SOCS1* [13]; CIMP as defined by this panel did not show a relationship to *KRAS*. Using quantitative real-time PCR, Ogino et al. [34] selected a panel of markers to distinguish high from low levels of methylation including *MLH1* and *CDKN2A*, and three markers that differ from the classic panel: *CACNA1G*, *CRABP1*, and *NEUROG1*. Shen et al. examined genetic markers (*BRAF, KRAS*) and epigenetic markers at 27 promoter-associated CpG sites using clustering analysis to identify two distinct CIMP+ groups: CIMP1, characterized by MSI+ and* BRAF* mutations, and MINT1, *MLH1, RIZ1, TIMP3* methylation; and CIMP2, characterized by *KRAS* mutations and methylation of several MINT markers [33]. 

Using structural equation modeling to construct causal models of CIMP and locus-specific CpG island methylation and a large database of colorectal cancers, Tanaka et al. showed the correlation structures of 16 methylation markers and CIMP status differed between *BRAF *mutated, *KRAS *mutated, and wildtype *BRAF*/*KRAS* tumors [35]. They suggested a possible role of these mutations differentially modifying the propensity for locus-specific methylation at the cellular level. To examine the question of whether or not *BRAF* V600E plays a causal role in the development of CIMP, Hinoue et al. determined 100 CIMP-associated CpG sites and examined changes in DNA methylation in eight stably transfected clones over multiple passages [38]. They observed that *BRAF *was not sufficient to induce CIMP in their system. 

In contrast to evaluation of relatively small sets of CIMP markers, comprehensive DNA methylation profiling and unsupervised hierarchical clustering were recently used to identify several CpG sites that were differentially methylated between tumor and normal tissue [36]. Using a similar approach, the use of two methylation panels as classifiers of colorectal cancer has been proposed: the first to identify highly methylated tumors (strongly correlated with *BRAF*) and a second to distinguish between intermediate (associated with *KRAS*) and low methylation groups [37]. Since epigenetic therapy is in clinical use or trials for several cancers, efficient methods for epigenetic profiling are needed; Kondo and Issa provide a summary of available profiling techniques and their features [39]. As approaches to CIMP characterization in colorectal cancer continue to evolve, it is clear that *BRAF* and* KRAS *oncogene mutations will continue to refine any definition of CIMP. Although characterization of CIMP status depends on methylation markers and criteria used, classification of tumors by both CIMP and MSI status recently proposed by Jass [40] and further refined by Ogino and Goel [14] has become an increasingly common strategy to define the pathological and clinical features of colorectal cancer.

## 4. Epidemiology of CIMP

### 4.1. Characteristics

Relationships between CIMP and clinicopathologic features of colorectal tumors that have been widely reported include proximal location, older age at diagnosis, female gender, poor tumor differentiation, MSI (CIMP high cancer), *BRAF *mutations*, KRAS* mutations (microsatellite stable cancer), and wildtype *TP53* [11, 13, 16, 17, 34, 41]. Based on a large Australian cohort, English et al. reported that individuals of southern European ethnicity had lower risk of CIMP and *BRAF* mutation than those with origins in northern Europe [21].

Using actual data and a classification tree method to visualize carcinogenic pathways, Slattery et al. suggested that unique mutational pathways to colon and rectal cancer likely exist [18]. This method describes the probability of developing various alterations in proximal colon, distal colon, or rectal tumors given previously acquired mutations. Using bootstrap resampling, the probabilities of developing specific mutations differed across tumor sites. For example, the estimated proportion of tumors that will develop methylation at CpG sites decreased as one goes from proximal colon to rectal cancers. Regardless of site, a methylation pathway in which *BRAF* is subsequently acquired independent of MSI or *MLH1* methylation was predicted. This work supports previous observations that link CIMP and *BRAF* mutation, independent of MSI status [11, 12].

### 4.2. Smoking

The presence of methylation in human malignancies bears a relationship to a history of cigarette smoking. Cigarette smoking has been associated with CpG island methylation within the bronchial epithelium of smokers and in lung cancer, and activation of the aromatic hydrocarbon receptor by cigarette smoke has been associated with CpG methylation [42–44]. A significant relationship has been reported between cigarette smoking and CIMP (and closely related mutations in *BRAF*) in colon and rectal carcinomas in both prospective cohort and case-control studies [45–48]. Interestingly, the relationship between smoking and CIMP provides an explanation for the previously observed association between cigarette smoking and MSI, as most of these tumors also exhibit CIMP [49]. Evidence also suggests that cigarette smoking (related to CIMP and *BRAF*) may be associated with hyperplastic polyps rather than adenomatous polyps; as mentioned above, a subset of hyperplastic polyps has been hypothesized to be the precursor to CIMP high colorectal carcinomas [50].

### 4.3. Other Risk Factors

S Adenosylmethionine (SAM), the universal donor of methyl groups in humans, and S Adenosylhomocysteine (SAH), the product of and an inhibitor of DNA methyltransferase (DNMT) enzymes, provide connections between folate metabolism and DNA methylation [51]. It has been hypothesized that diets low in folate and high in alcohol intake may disturb DNA methylation, which may result in global DNA hypomethylation concurrently with a greater risk of cancers with CpG island methylation [52, 53]. In contrast, other studies have shown that global DNA hypomethylation is inversely correlated with CIMP and may represent different pathways to colorectal cancer [54, 55]. Studies generally do not support a unique role for alcohol and folate in CIMP+ tumors [56, 57], although genetic polymorphisms in *MTHFR* 1298A > C (rs1801131), interacting with diet, and *TCN2* 776G > C (rs1801198) may be involved in the development of highly CpG-methylated colon and rectal cancers [58–60]. Conversely, *MTHFR* 1298A > C was not associated with CIMP+ tumors in the Netherlands cohort study [61]. Polymorphisms in DNA repair genes have been implicated in CIMP-positive colon cancer [62, 63]. A promoter polymorphism in *MLH1* (−93G > A) was associated with CIMP, *MLH1* methylation, and *BRAF* mutations in unstable sporadic colon tumors and not in stable tumors, suggesting the genetic variant may be acting at a relatively late stage of carcinogenesis to drive CIMP-positive tumors down the microsatellite instability pathway [63].

Overexpression of DNMT3B has been shown to be a risk factor for the development of CIMP in colorectal cancer [64, 65]. DNMT3B is important in establishing and maintaining genomic methylation patterns, and overexpression in mice can induce tumors with methylation in specific CpG islands. Recent findings indicate that DNA methylation changes occur sequentially during tumor progression, and DNMT3B expression levels increase during this progression [66]. 

The future of CIMP in colorectal cancer research may well take place in the evolving trans- and interdisciplinary field of “molecular pathological epidemiology” outlined by Ogino et al, which is designed to elucidate how genetic factors and lifestyle exposures interact with specific molecular subtypes of cancer [67]. Hughes et al. reported that severe caloric restriction was associated with decreased risk of developing a tumor characterized by CIMP. This study provides a potential link between early life conditions and epigenetic changes that later influence colorectal cancer development [68]. The work of Slattery et al. regarding differences in the etiologies of rectal-site and colon-site tumors, and the influence of genetic factors in the inflammatory pathway in the etiology of CIMP in both, is an example of this approach [69, 70].

## 5. Emerging Trends in CIMP Research

Although aberrant DNA methylation of promoter CpG islands in cancer genes as well as repressive chromatin are frequently involved in gene inactivation during tumorigenesis, the mechanisms underlying CIMP are poorly understood. Patterns of hypermethylation are specific to tumor type, and it is unclear why certain regions become hypermethylated; however, mapping of the human methylome as a result of technological advances has expanded our understanding of epigenetic mechanisms [71]. Inactivation of particular genes may confer a growth advantage, resulting in clonal selection [72]. Another possibility is that long-range epigenetic silencing by DNA methylation can span chromosome regions of 1 Mb in colorectal cancer, resembling the loss of heterozygosity often observed in tumors [73]. In a large cohort of sporadic colorectal cancers, Wong et al. reported a strong relationship between long-range silencing of chromosome region 3p22 and CIMP+ tumors [74].

Recent findings suggest that most DNA methylation alterations in colon cancer occur in CpG island shores, sequences up to 2 kb distant from CpG islands [75]. Hypermethylated CpG shores appear closer to their associated CpG islands, while hypomethylated shores occur further away from their associated islands and resemble noncolon normal tissues. These findings are consistent with an epigenetic progenitor model of cancer, in which epigenetic alterations affecting tissue-specific differentiation are the predominant mechanism by which epigenetic changes cause cancer. Alternative transcription may be a function of differential DNA methylation during differentiation, and one role of altered methylation in cancer may be to disrupt regulatory control of specific promoter usage [75].

Previous studies suggest a general model in which genes reposition away from the heterochromatin when activated and gravitate to heterochromatin when silenced [76]. However, Easwaran et al. demonstrated that aberrant silencing of cancer-related genes occurred without requirement for their being positioned at heterochromatic domains using immunostaining for active/repressive chromatin marks and fluorescence in-situ hybridization in CRC cell lines. Furthermore, CpG hypermethylation, even associated with long-range silencing of nearby genes, occurred independently of their heterochromatic or euchromatic location [77]. These findings have important implications for understanding relationships between gene expression patterns and nuclear organization in cancer.

Another area under investigation is the understanding of mechanisms underlying which tumor suppressor genes are targeted for inactivation in cancer. Studies suggest a stem cell origin linked to epigenetic control of gene expression patterns in precursor cells regulated by constituent proteins in PcG repressive complexes including Polycomb Repressive Complex PRC1 [78, 79]. It was subsequently shown that sustained expression of the PRC1 protein, CBX7 along with other proteins, targets gene promoters in a progenitor-like embryonic tumor cell resulting in a cell population that models epigenetic characteristics of adult cancer (including aberrant CpG-island methylation) via inhibited response to differentiation cues [80]. 

DNA methylation markers have potential clinical use as diagnostic and prognostic tools. Hypermethylation of CpG islands can serve as a biomarker of cancer cells in tumor biopsies and other specimens. For example, quantitative assessment of methylation in CIMP-specific promoters of *MLH1*, *WRN*, and other DNA-repair genes in colon tumors, in comparison to paired normal tissues, may predict response to treatment [2]. Profiles of miRNA expression also differ between tumor and normal tissues; silencing of *miR-124a* in colon cancer cells activates expression of the oncogene *CDK6* [5]. Continued research involving detailed DNA methylomes in healthy and diseased tissues will help distinguish causal epigenetic alterations from “bystander changes” which are a consequence of cellular processes [71].

Unlike mutations in DNA sequence, epigenetic alterations such as CpG Island hypermethylation are potentially reversible by “reawakening” silenced tumor suppressor genes. Two nucleoside DNA methylation inhibitors, azacitidine and decitabine, are used clinically in low doses to treat myelodysplastic syndrome, providing proof of principal for epigenetic therapy [81]. Structurally, these agents mimic cytosine; during cell replication, fake cytosines replace real cytosines in growing DNA strands and then trap DNA methyltransferases to interfere with the ability of these enzymes to reproduce existing methylation in new cells. While inhibiting DNA methylation is a targeted molecular approach to therapy, downstream effects on neoplastic behavior are nonspecific and may result in cytotoxic cell death, making predictions of clinical outcomes difficult [81]. Clinical trials are being extended to test DNMT inhibitors in solid tumors of the breast, lung, and colon, in combination with histone deacetylase (HDAC) inhibitors which provide synergistic benefits in cell studies [82]. Other treatment avenues on the horizon include induced cellular programming to guide development of epigenetic-modifying drugs [83]. 

**Figure 2:**
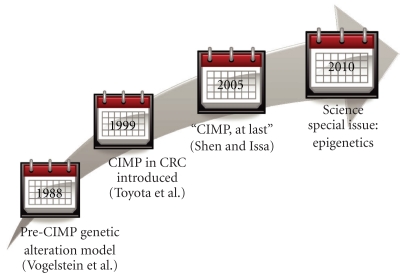
A decade of epigenetic research in colorectal cancer (CRC) has led to widespread recognition and acceptance of the CpG Island Methylator Phenotype.

It has been a little over a decade since the concept of a CpG island methylator phenotype in colorectal cancer was introduced, and subsequent focus of several studies on describing the clinical and pathological features of CIMP as well as its characterization in tumors has supported widespread acceptance of the role of DNA methylation in cancer (Figure 2). The past few years have brought substantial insights as to the mechanisms underlying the CIMP pathway in cancer, and the future development of diagnostics and treatments based on our understanding of this epigenetic alteration are an exciting development in epigenetic research.

## Figures and Tables

**Table 1:** A history of CIMP panels used to assess CpG island methylation in colorectal cancer.

Study	CIMP panel markers	Notes
Toyota et al. [7]	*CDKN2A (p16)*, MINT1, MINT2, MINT12, MINT17, MINT25, MINT27, MINT31, *MLH1, THBS1 *	Pioneering work to identify markers that distinguish CIMP from age-related methylation
Park et al. [31]	*CDKN2A*, MINT1, MINT2, MINT31, *MLH1 *	So-called “classic” or traditional panel
Weisenberger et al. [13]	*CACNA1G, IGF2, NEUROG1, RUNX3, SOCS1*	“New” panel based on stepwise screen of 195 markers
Ogino et al. [34]	*CACNA1G, CDKN2A, CRABP1, MLH1, NEUROG1*	Selected markers to distinguish high-level from low-level methylation
Shen et al. [33]	CIMP1: MINT1, *MLH1, RIZ1, TIMP3, BRAF *mutation; CIMP2: MINT2, MINT27, MINT31, Megalin, *KRAS *mutation	Examined 27 CpG sites, proposed optimal epigenetic and genetic markers to identify CIMP1, CIMP2, or CIMP-
Tanaka et al. [35]	*CACNA1G, CDKN2A, CHFR, CRABP1, HIC1, IGF2, IGFBP3, MGMT*, MINT1, MINT31, *NEUROG1, p14, RUNX3, SOCS1, WRN *	Correlation structures of markers and CIMP differ by *KRAS *and *BRAF *status
Ang et al. [36]	Total of 202 CpG sites differentially methylated between tumor and normal	Comprehensive DNA methylation profiling in 807 cancer genes
Kaneda and Yagi [37]	Group 1: *IGF2, LOX*, MINT1, MINT2, MINT31, *MLH1, RUNX3, SOCS1;* Group 2: *ADAMTS1, DUSP26, EDIL3, ELMO1, FBN2, HAND1, IGFBP3, NEUROG1, RASSF2, STOX2, THBD, UCHL1 *	Comprehensive DNA epigenotyping of genomewide regions indentified two groups (high and intermediate to low methylation)
